# Tuberculous Meningitis in Adults: A Review of 160 Cases

**DOI:** 10.1100/2012/169028

**Published:** 2012-04-24

**Authors:** Filiz Pehlivanoglu, Kadriye Kart Yasar, Gonul Sengoz

**Affiliations:** Department of Infectious Diseases and Clinical Microbiology, Haseki Training and Research Hospital, Istanbul 34300, Turkey

## Abstract

*Objective*. This study aimed to evaluate epidemiological, clinical, laboratory, and neuroimaging features of 160 adult patients with tuberculous meningitis (TBM) according to “Thwaites' diagnostic index.” *Methods*. The subjects of this retrospective study are the patients with TBM who were followed up between years 1998 and 2009 in a tertiary referral hospital. Diagnosis of TBM was based on clinical, laboratory, and neuroimaging signs and Thwaites' diagnostic index. *Results*. *Mycobacterium tuberculosis* was isolated from CSF in 59 of 148 patients. Seventeen percent of the patients died, 71% recovered completely, and 13% recovered with neurological sequel at the end of the sixth month. *Conclusions*. Despite new developments in laboratory or neuroimaging techniques, the diagnosis of TBM is still based on clinical features with the help of laboratory. Early diagnosis by suspecting TBM may prevent therapy delay and may result in decrease in the mortality and morbidity.

## 1. Introduction

Tuberculosis (TB) is a disease which has been affecting humanity since archaic ages. Although tuberculous meningitis (TBM) is the least commonly observed form of extrapulmonary TB (5–15%), it is the most severe form in terms of mortality and morbidity [1–3]. It develops as an early or late complication of primary infection. Mortality is frequently associated with a delay in diagnosis and treatment [4]. Clinicians involved with TBM are faced with serious problems regarding both the diagnosis and the treatment of the disease. Question marks still remain as to the pathogenesis of the disease; quick and reliable diagnostic tests are not available to be used at early stage; some patients could still die or be left disabled despite effective treatment. There are globally accepted criteria on TBM used by clinicians to aid diagnosis [5–7].

Our objectives in this study are (1) to review the clinical, laboratory, and radiological findings from 160 TBM cases diagnosed, treated, and followed up at a single center in the light of the literature, (2) to present the local epidemiological data from our cases, and (3) to highlight the results from our cases and emphasize the significance of the Thwaites' diagnostic index, which we consider a practical and easy-to-implement guide.

## 2. Methods

The cases in the study were patients older than 14 years of age who were diagnosed with TBM at our hospital during the 11-year period between January 1998 and March 2009. Clinical, laboratory, and radiological characteristics of the cases, which were obtained through the retrospective review of hospital files and outpatient follow-up files, were summarized by using a standard data collection table. Cases with incomplete data or uncertain diagnosis were excluded from the study.

 TBM was diagnosed based on the clinical characteristics, laboratory, cerebrospinal fluid (CSF) findings, and radiological imaging methods. Cases suffering from fever and headache for two weeks or more, with a stiff neck or altered sensorium detected in physical examination, and with lymphocytic pleocytosis, decreased glucose, and increased protein levels detected in CSF examination, were considered lymphocytic meningitis. Lymphocytic meningitis cases with these characteristics were considered TBM in the presence of the following additional criteria: (1) detection of acid-resistant bacteria in CSF, other sterile body fluids, tissue under direct microscopy with EZN staining, or growth of such bacteria in culture, (2) demonstration of the DNA of *M. tuberculosis* in CSF or in other sterile body fluids or in tissue with PCR, (3) close or familial contact with an active pulmonary TB case, (4) prior TB or family history of TB, (5) characteristic findings suggesting TB in cranial imaging (basal meningitis, tuberculoma, etc.), (6) presence of pulmonary TB findings such as active infiltration, miliary pattern, or cavity in pulmonary imaging, and (7) clinical response to antituberculosis therapy (ATT). In addition, Thwaites' diagnostic index was used retrospectively for cases followed up until 2002, prospectively for cases followed up between 2002 and 2009, and cases with a score value ≤4 were considered TBM [8]. The cases are revised based on standardized clinical case definition that was mentioned in the 2010 article of Marais. The criteria used in classification of Marais are as follows:

1. clinical criteria (maximum category score = 6),
2. CSF criteria (maximum category score = 4),
3. cerebral imaging criteria (maximum category score = 6),
4. evidence of tuberculosis elsewhere (maximum category score = 4).

All cases were classified as definitive, probable, possible, or not tuberculous meningitis, depending on their total diagnostic score. Definite tuberculous meningitis: it is the microbiological identification or evidence from commercial nucleic acid amplification tests of CNS *M. tuberculosis* infection. 59 cases fit this definition. Probable tuberculous meningitis: when imaging is available, a diagnostic score of 12 or above is required (28 cases), and when imaging is not available, a diagnostic score of 10 or above is required (12 cases). Total 40 cases fit this definition. Possible tuberculous meningitis: when imaging is available, a diagnostic score of 6–11 is required (56 cases), and when imaging is not available, a score of 6–9 is required (4 cases). Total 60 cases fit this definition. One case was excluded from the study in which lumbar puncture and imaging are not possible [7].

The cases were clinically evaluated and neurologically staged during admission to the hospital based on the British Medical Research Council criteria [9]. Accordingly, mild cases with nonspecific symptoms that were conscious and did not have neurologic deficits were considered stage I; cases with mild alterations in consciousness and minor neurologic deficits such as cranial nerve palsies were considered stage II; while cases with major neurologic deficits such as paresis/plegia, cases with convulsions, and cases in precoma/coma state were considered stage III.

The cases were treated with the classical four-drug ATT (combination of izoniazid-INH, rifampicin-RIF, pirazinamide-PRZ, and ethambutol-EMB) for 12–18 months. Some cases with prior TB received a five-drug therapy including streptomycin. Cases with antituberculosis resistance received a combination therapy including minor drugs. Advanced stage cases with neurological findings were also given intravenous dexamethasone therapy for eight weeks.

## 3. Results

The 160 TBM cases included in the study were aged 14–78 with a mean age of 32.18 ± 13.62. Half of the cases were female. At the time of admission to the hospital, only 16% of the cases were stage I, while 84% were stage II and stage III. All cases were tested for HIV/AIDS, and only one male had positive HIV serology. Two cases were pregnant, and one case had given birth four months ago. The demographic, clinical, laboratory, and radiological findings of the cases are summarized in Table 1.

 The symptom duration of the cases varied between two and 365 days. The time between the onset of complaints and application to the hospital was less than one week in 11 cases (7%), 1–3 weeks in 91 cases (57%), and more than three weeks in 58 cases (36%). This duration was longer than six months in two cases and 12 months in one case. 

 The most frequent symptoms observed at the time of admission were headache (86%), nausea vomiting (64%), and altered sensorium (59%). While the most frequent finding was stiff neck (88%), meningeal irritation was detected only in 37% of the cases. During initial admission, 24% of the cases had cranial nerve palsies, 21% were in precoma/coma state, and 16% had convulsions. Abducens nerve palsy was the most frequently observed cranial nerve palsy, followed by oculomotor and facial nerve palsies. Four cases had oculomotor, and four had acoustic nerve palsies. 

According to the Thwaites' diagnostic index (Table 2), all cases had a score of ≤4, and 70% had a score of (−5), which is the most significant value for TBM diagnosis. 

38% of the cases had accompanying extraneural TB, primarily pulmonary TB. Extraneural TB cases comprised pulmonary TB in 47 cases, Pott's disease in six cases, renal TB in two cases, and gastrointestinal and skin TB in one case each. In addition, 43 cases (27%) had prior TB, and 31 cases had family history of TB (19%). 37 cases (23%) had underlying diseases including diabetes mellitus (10), trauma (6), pregnancy/delivery (4), alcoholism (4), malignity (3), convulsions (2), idiopathic thrombocytopenic purpura (1), mental retardation (1), SLE (1), multiple sclerosis (1), herpes zoster (1), cerebrovascular disease (1), vitiligo (1), and HIV/AIDS. 

*M. tuberculosis* was isolated from CSF in 59 (40%) of the 148 cases who could be subject to lumbar puncture and from other body parts in 7 cases in the Lowenstein-Jensen medium. Blood WBC counts were 1,500–32,800/mm^3^ and within normal limits in 97 cases (61%) (4,000–10,000/mm^3^). Erythrocyte sedimentation rate was increased in 75% of the cases (66/87); positive and anergic reactions were observed in 33% and 49% of the 43 cases tested with PPD, respectively. 

In CSF examination, mean leukocyte count was found to be 233/mm^3^(1–2290/mm^3^), and lymphocyte domination was detected in 65%. CSF/serum glucose ratio was <0.6 and <0.3 in 95% and 55% of the cases, respectively. CSF protein was normal in only 13 cases and >100 mg/dL in 72% of the cases. 

**Figure 1:**
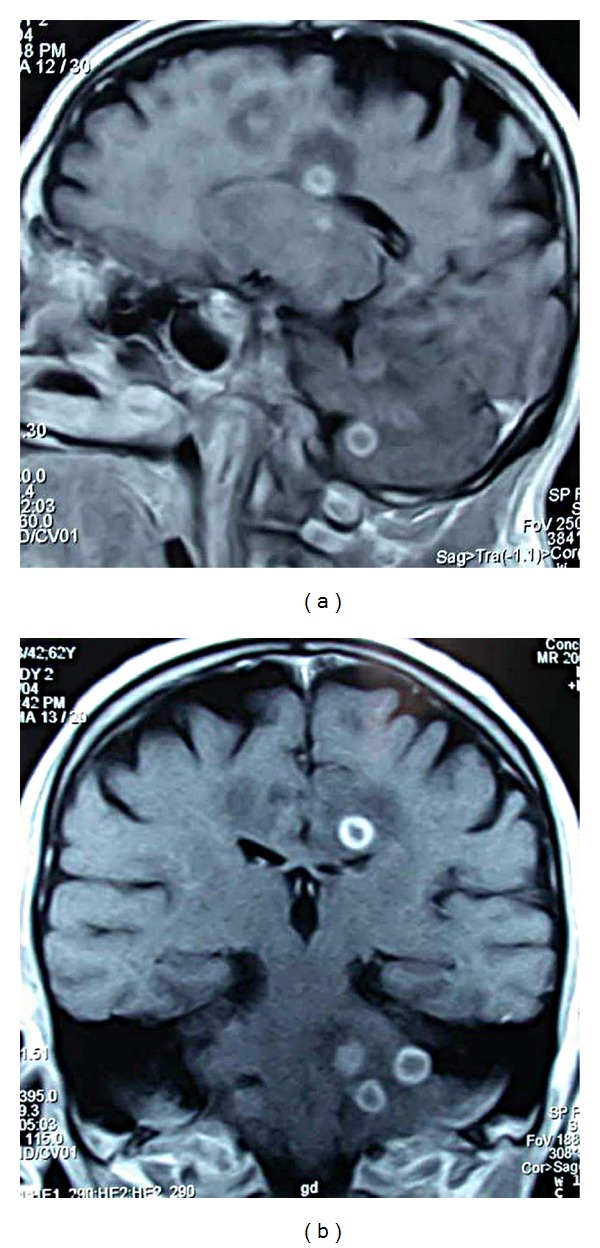
Ring-like contrast-enhancing multiple tuberculomas with a hypointense center and perifocal edema in the posterior fossa, frontal lobe, and around corpus callosum in T1W coronal and sagittal plane as shown with contrast-enhanced cranial MRI.

 TB-associated findings were observed in the chest X-rays of 73 of the 101 cases (72%). 25 cases had miliary pattern, 35 had active parenchymal infiltration, seven had cavitary lesions, and six had pleurisy. Of the cases on which cranial imaging could be performed (136/160; 85%), only contrast CT was used in 32 cases; only contrast MRI was used in 75 cases, and combination of CT and MRI was used in 27 cases. The most frequently identified cranial radiological findings were tuberculoma (in 49 cases; 37%), basal meningitis (in 36 cases; 27%), hydrocephalus (in 28 cases; 21%), and ischemia/infarct (in 12 cases; 9%) (Figure 1). 

ATT and dexamethasone therapy were started between the 1st and 33rd days of hospitalization. Therapy was started within the first 3 days in 76% of the cases, within 4–10th days in 18%, and on the 33rd day in one case. 92% of the cases were given the classical four-drug therapy consisting of INH, RIF, PRZ, and EMB, while 6% of the cases received a five-drug therapy including streptomycin. During hospitalization, 56% of the cases recovered completely, 34% recovered with neurological sequelae, and 10% died. During the six-month follow-up period, these rates changed to 71%, 13%, and 17%, respectively. 

Majority of the dying cases died within the first ten days, and 13 of the 27 cases had an underlying disease (diabetes, trauma, alcoholism, etc.). The mean age of the dying cases was 45; 16 of them were stage III and 11 were stage II. Of the dying cases, seven were in coma state at the time of admission to the hospital, two had altered sensorium, and two had a neurological deficit. Two cases died on the day of admission to hospital, two died on the 3rd day before therapy could be started, and in two cases, ATT was started on days 24 and 33 following hospitalization. 10 of the dying 27 cases had leukocytosis. 

## 4. Discussion 

Before *M. tuberculosis* was identified by Robert Koch in 1882, TBM was clinically described by Robert Whytt in 1762 for the first time in children with acute hydrocephalus [10]. Until the discovery of antituberculosis drugs in the second half of the 20th century, TBM was a fatal disease for everyone. However, its mortality can still reach 60% today particularly in developing countries. Sequelae can be seen in 25% of survivors despite five major and numerous minor drug options available [11, 12]. As advanced disease stage and delay in therapy are considered poor prognostic factors, early diagnosis and treatment is important. 

TBM is most frequently seen as a complication of the primary infection during the first five years of life in countries with a high incidence of TB, while it basically develops through the reactivation of meningeal or subcortical focus in adults aged 25–45 in countries with a low incidence. In large trials conducted in Turkey, young adults aged 15–30 have been reported as the group with the highest incidence rate [13, 14]. In our study, 53% of the cases were aged 15–30, 47% were older than 30, and 14% were older than 45 years old. A large metropolis with a higher TB incidence (56/100,000) than the overall incidence in Turkey (29/100,000), Istanbul is home to both a young population with poor social and economic conditions and an old population with over-the-average living conditions. Therefore, cases from both young and old population were included in our study [15]. Nearly 60% of the cases reported in the publications in our country comprise males [2, 4, 16]. In our study, the ratio of females to males is 1 : 1, which is considered to be consistent with the dominance of women in extrapulmonary TB cases versus the male dominance in pulmonary cases [17–19]. 

During hospital admission, 63% of the cases were stage II, and 21% were stage III. At this time, 59% of the cases had altered sensorium, 16% had convulsions, 21% were in a coma state, and 15% had paresis/plegia, all of which were neurologically serious cases as in the study by Sütlaş et al. [20]. However, this was not due to an accompanying HIV infection or malnutrition. Only one of the cases was HIV positive; yet 23% had an underlying disease such as diabetes, trauma, malignancy, and alcoholism. Particularly, factors such as diabetes, malignancy, and alcoholism are known to play a role in TBM development; therefore, they can be considered to contribute to a poor prognosis as well [21]. However, the mortality rate in our study was lower than other reports in Turkey although the cases were severe and in advanced stage [4, 14, 16, 20]. This could be associated with the fact that each case was treated with at least a four-drug ATT, and an early and long treatment strategy was administered. Each case was given at least a four-drug protocol consisting of INH, RIF, PRZ, and EMB for 12–18 months. In addition, treatment was started within the first three days following hospitalization in 76% of the cases, and within 4–10 days in 18%. Of the dying cases, treatment was even started within the first three days in 20 cases except the four cases that died before treatment could be started and two cases where the initiation of therapy was delayed. Therefore, poor prognostic factors other than the delay in treatment are thought to contribute to patient mortality. Besides, the rate of sequelae identified during the six-month follow-up period was 13%, which was found to be lower than the other reports in Turkey [14, 20]. It was noteworthy that the cases developing sequelae were young and had cranial nerve palsies at the time of admission. It was also demonstrated in the study by Hoşoğlu et al. [4] that cranial nerve palsy at the time of hospital admission was a risk factor for the development of neurological sequelae. 

TBM is both an insidious disease, and it can have an atypical clinical picture. Identification and monitoring of patients according to standard case definitions are important in providing comparable results among different studies. Therefore, suspecting TBM could save lives in cases with nonspecific symptoms such as long-lasting fever, weight loss, and adynamia in the presence of clinical signs like headache, nausea vomiting, and absent mindedness, which are suggestive of intracranial pressure increase if there is also accompanying pulmonary TB, family history of TB, or close contact with an active TB case. Headache, fever, and nausea vomiting were the most frequently observed complaints in our study. Thwaites' diagnostic index scoring was reported to be a practical, precise, and specific that can be used to differentiate TBM from bacterial meningitis based on age, disease or complaint duration, WBC count, and CSF findings [8, 22, 23]. In our study, all cases had a score of ≤4, which is the limit value for TBM according to this Index, and 70% had the maximum score which is significant for TBM. The golden standard for TBM diagnosis is the isolation of the disease agent, but this is not always possible. While this rate is higher abroad, *M. tuberculosis* has largely been isolated in a maximum of half of the cases reported in Turkey. In our study, TB culture positivity in CSF (40%) was found to be higher than the other reports in our country [4, 16, 24, 25]. 

The cases in the study were diagnosed based on clinical, laboratory, and radiological findings. Typical CSF findings, family history of TB, close contact with an active TB case, and prior TB in addition to high culture positivity aided the diagnosis. Additionally, the presence of accompanying extraneural TB, primarily pulmonary TB, and characteristic findings of TB in cranial imaging in 38% of the cases once again demonstrates the importance of radiology for TBM diagnosis. Of the 136 cases on which cranial imaging could be performed, combination of CT and MRI was used in 27 cases, and positive findings in MRI were identified in eight of the 13 cases that are found to be normal with CT. The results from our study, where a higher number of basal meningitis, tuberculoma, leptomeningeal palsy and infarct cases could be demonstrated with MRI, are consistent with the publications stressing the superiority of MRI to CT [26, 27]. In the study by Piennar et al. [27] conducted on children with TBM, MRI was also found to be more useful than CT for the estimation of poor outcomes in addition to its diagnostic superiority. 

BCG is in children vaccination schedule in Turkey. Therefore, PPD positivity is not useful in diagnosis. However, since interferon-gamma release tests, that are more widely in use in recent years, do not have cross-reaction with BCG; they are more helpful in diagnosing latent TB. 

Consequently, as in every form of TB, TBM is still a major health issue in Turkey. The diversity of its clinical findings and the lack of practical and reliable methods for early diagnosis encourage the clinicians involved with the disease to create diagnostic guidelines to reduce mortality and morbidity and to spread the use of such guidelines [7, 24]. Therefore, suspecting TBM and starting treatment early could save lives in cases with the clinical signs and symptoms of meningitis, and in cases with history and risk factors of tuberculosis. Standardized case definitions will make the conclusions of the researchers, who review previously published papers, more valuable.

## Figures and Tables

**Table 1:** Clinical and laboratory characteristics of TBM cases.

		*n* (%)
Headache		138 (86.3)
Fever		110 (69.2)
Nausea vomiting		102 (63.8)
Lack of appetite weakness		65 (40.6)
Change in personality		44 (27.5)
Weight loss		42 (26.3)
Night sweats		37 (23.1)
Stiff neck		141 (88.1)
Meningeal irritation findings		59 (36.9)
Blurred consciousness		95 (59.4)
Cranial nerve palsy		38 (23.8)
Coma		33 (20.6)
Convulsions		25 (15.6)
Plegia/paresis		24 (15)
		
Peripheral WBC count (*n* : 131)	normal*≠*	97 (74)
	abnormal*	34 (26)
CSF WBC count/mm^3^ (*n* : 148)	<100	47 (31.8)
	100–500	86 (58.1)
	>500	15 (10.1)
CSF/blood glucose ratio (*n* : 148)	<0.60	140 (94.6)
	≤0.30	81 (54.7)
CSF protein level mg/dL (*n* : 148)	<40	13 (8.8)
	40–150	74 (50)
	>150	61 (41.2)
Culture positivity in CSF		59 (39.9)
		
Cranial CT or MRI *n* : 134	Tuberculoma	49 (36.6)
	Basal meningitis	36 (26.9)
	Leptomeningeal palsy	34 (25.4)
	Hydrocephalus	28 (20.9)
	Edema	16 (11.9)
	Ischemia-infarct	12 (8.9)
	Abscess	5 (3.7)
	Arachnoiditis	3 (2.2)
	Normal	30 (22.4)

Normal *≠* 4000–10000/mm^3^.

Abnormal* >10000/mm^3^.

**Table 2:** Thwaites' diagnostic index [8].

Characteristic		Index
Age	≥36 <36	2 0
WBC	≥15000/mm^3^ <15000/mm^3^	4 0
Complaint duration	<6 days ≥6 days	0 −5
BOS WBC	≥900/mm^3^ <900/mm^3^	3 0
BOS WBC % PNL	≥75 <75	4 0

## References

[1] World Health Organization, Global Tuberculosis Database http://www.who.int/research/en/.

[2] Bozluolcay M, Pelin Z (2003). Tuberculosis of the central nervous system in Turkey: a retrospective study of 90 adult patients. *Journal of Neurological Sciences*.

[3] Porkert MT, Sotir M, Parrott-Moore P, Blumberg HM (1997). Tuberculous meningitis at a large inner-city medical center. *American Journal of the Medical Sciences*.

[4] Hoşoğlu S, Geyik MF, Balik I (2002). Predictors of outcome in patients with tuberculous meningitis. *International Journal of Tuberculosis and Lung Disease*.

[5] Thwaites G, Chau TTH, Mai NTH (2000). Tuberculous meningitis. *Journal of Neurology Neurosurgery and Psychiatry*.

[6] Thwaites G, Fisher M, Hemingway C, Scott G, Solomon T, Innes J (2009). British Infection Society guidelines for the diagnosis and treatment of tuberculosis of the central nervous system in adults and children. *Journal of Infection*.

[7] Marais S, Thwaites G, Schoeman JF (2010). Tuberculous meningitis: a uniform case definition for use in clinical research. *The Lancet Infectious Diseases*.

[8] Thwaites GE, Chau TTH, Stepniewska K (2002). Diagnosis of adult tuberculous meningitis by use of clinical and laboratory features. *The Lancet*.

[9] MRC (1948). Streptomycin treatment of tuberculous meningitis. *British Medical Journal*.

[10] Zuger A, Scheld WM, Whitley RJ, Marra CM (2004). Tuberculosis. *Infections of the Central Nervous System*.

[11] Kent SJ, Crowe SM, Yung A, Lucas CR, Mijch AM (1993). Tuberculous meningitis: a 30-year review. *Clinical Infectious Diseases*.

[12] Thwaites GE, Bang ND, Dung NH (2004). Dexamethasone for the treatment of tuberculous meningitis in adolescents and adults. *The New England Journal of Medicine*.

[13] Sengoz G, Yasar KK, Yildirim F (2008). Evaluation of 121 adult cases of tuberculous meningitis. *Neurosciences*.

[14] Hoşoğlu S, Geyik MF, Balik I (2003). Tuberculous meningits in adults in Turkey: epidemiology, diagnosis, clinic and laboratory. *European Journal of Epidemiology*.

[15] Tuberculosis control in Turkey, 2007 report

[16] Avci M, Ozgenç O, Arı A (2007). Evaluating cases of tuberculous meningitis. *Turkish Journal of Infection*.

[17] Peto HM, Pratt RH, Harrington TA, LoBue PA, Armstrong LR (2009). Epidemiology of extrapulmonary tuberculosis in the United States, 1993-2006. *Clinical Infectious Diseases*.

[18] Lin JN, Lai CH, Chen YH (2009). Risk factors for extra-pulmonary tuberculosis compared to pulmonary tuberculosis. *International Journal of Tuberculosis and Lung Disease*.

[19] Abuaku BK, Tan H, Li X, Chen M, Huang X (2010). A comparative analysis of tuberculosis treatment success between hunan province of China and Eastern Ghana. *Medical Principles and Practice*.

[20] Sütlaş PN, Ünal A, Forta H, Şenol S, Kirbaş D (2003). Tuberculous meningitis in adults: review of 61 cases. *Infection*.

[21] Kaptan F (2005). Tuberculous meningitis. *Turkish Journal of Infection*.

[22] Sunbul M, Atilla A, Esen S, Eroglu C, Leblebicioglu H (2005). Thwaites’ diagnostic scoring and the prediction of tuberculous meningitis. *Medical Principles and Practice*.

[23] Török ME, Nghia HDT, Chau TTH (2007). Validation of a diagnostic algorithm for adult tuberculous meningitis. *American Journal of Tropical Medicine and Hygiene*.

[24] Sengöz G (2005). Evaluating 82 cases of tuberculous meningitis. *Tuberkuloz ve Toraks*.

[25] Göktaş P, Ceran N, Coşkun D, Hitit G, Karagül E, Özyürek S (1998). Evaluation of 38 adult cases of tuberculous meningitis. *Klimik Journal*.

[26] Chan KH, Cheung RTF, Fong CY, Tsang KL, Mak W, Ho SL (2003). Clinical relevance of hydrocephalus as a presenting feature or tuberculous meningitis. *Quarterly Journal of Medicine*.

[27] Pienaar M, Andronikou S, Van Toorn R (2009). MRI to demonstrate diagnostic features and complications of TBM not seen with CT. *Child’s Nervous System*.

